# Single administration of intra-articular bupivacaine in arthroscopic knee surgery: a systematic review and meta-analysis

**DOI:** 10.1186/s12891-015-0477-6

**Published:** 2015-02-10

**Authors:** Qi-Bin Sun, Shi-Dong Liu, Qin-Jun Meng, Hua-Zheng Qu, Zheng Zhang

**Affiliations:** Department of Spine and Joint Surgery, The Third People’s Hospital of Jinan, No.1 North Industrial Road, Wangsheren North Street, Jinan, 250101 Shandong People’s Republic of China

**Keywords:** Arthroscopic surgery, Bupivacaine, Efficacy, Safety, Meta-analysis

## Abstract

**Background:**

Single administration of intra-articular (IA) bupivacaine for pain relief after arthroscopic knee surgery is effective, but its active duration and dose–response relationship is unclear. We conducted this meta-analysis to summarize all published randomized controlled trials (RCTs), thus providing the most recent information on the safety and efficacy of single-administration IA bupivacaine for pain relief after arthroscopic knee surgery, and to determine whether a dose–response relationship exists.

**Methods:**

A systematic electronic literature search (through April 2014) was conducted to identify those RCTs that addressed the safety and efficacy of a single administration of IA bupivacaine for pain management after arthroscopic knee surgery. Subgroup analysis was conducted to determine changes in visual analog scale (VAS) scores at seven postoperative time points. Meta-regression and subgroup analyses were carried out to assess the effects of various treatment factors on efficacy and to evaluate the dose–response relationship of bupivacaine. Weighted mean differences or relative risks were calculated and pooled using a random-effects model.

**Results:**

Twenty-eight trials involving 1,560 patients who underwent arthroscopic knee surgery met the inclusion criteria. The trials were subject to medium risk of bias. VAS scores at 2, 4, 6, 12, and 24 h postoperatively were significantly lower, the number of patients requiring supplementary analgesia was smaller, and the time to first request for analgesia was longer in the IA bupivacaine group than in the placebo group. The analgesic effect of single-administration IA bupivacaine may be associated with the effect of concomitant administration of epinephrine and concentration of bupivacaine, and no dose–response relationship was identified. No significant difference in side effects was detected between groups.

**Conclusions:**

Current evidence shows that the use of single-administration IA bupivacaine is effective for postoperative pain management in patients undergoing arthroscopic knee surgery, with satisfactory short-term safety. Low-dose administration of IA bupivacaine 0.5% combined with epinephrine adjuvant in clinical practice should be performed. Additional high-quality RCTs with longer follow-up periods are required to examine the safety of single-administration IA bupivacaine.

**Electronic supplementary material:**

The online version of this article (doi:10.1186/s12891-015-0477-6) contains supplementary material, which is available to authorized users.

## Background

Arthroscopic knee surgery is one of the most common orthopedic procedures performed in the outpatient setting. Approximately 985,000 Americans underwent this surgery in 2006, according to national estimates [1]. Although knee arthroscopy causes less trauma than open surgery, considerable postoperative pain can hinder a patient’s ability to participate in early rehabilitation and affect the patient’s activity level and satisfaction. Patients are usually discharged shortly after surgery and must be provided with analgesia that is both safe and effective. Single intra-articular (IA) administration of local anesthetic has been used to provide better analgesia after arthroscopic knee surgery and to reduce consumption and possible side effects of oral and intravenous anesthetics.

Bupivacaine is often used for IA analgesia because of its extended period of active effectiveness [1]. The analgesic efficacy of IA bupivacaine, especially single-administration bupivacaine, has been studied because its effect on postoperative pain is conceptually simple. However, there are conflicting reports on the efficacy of single-administration IA bupivacaine. Despite reports supporting its use, the results of a number of studies on the efficacy of single-administration IA bupivacaine after arthroscopic knee surgery have been equivocal [2,3].

The active duration of analgesia provided by a single administration of IA bupivacaine is controversial as well. In a review by Moiniche *et al.* [4], the reduction in pain scores attributable to bupivacaine was short in duration, and some studies [5,6] have demonstrated bupivacaine to be superior to placebo for the first 2–4 h only. In contrast, other studies [7,8] showed bupivacaine to have a longer analgesic effect than placebo and to be more effective in the first 24 h. In other studies [3,9], bupivacaine dose and concentration were increased and augmented with epinephrine to obtain a longer analgesic effect. However, these findings were inconsistent, and a dose-dependent relationship with effectiveness could not be demonstrated [4]. Because of the simplicity and apparent safety of the technique, it has gained widespread acceptance and use; nevertheless, the safety of IA bupivacaine has also been questioned [10,11].

A recent meta-analysis by Wei *et al.* [1] indicated that single-dose intra-articular bupivacaine is better than placebo at relieving pain after arthroscopic knee surgery. However, the data from several RCTs were not included in their analysis, and because VAS scores were acquired at different time points in different included studies, the results were pooled (i.e., VAS scores from 24 h postoperatively in one study and 48 h postoperatively in another were combined). A high degree of heterogeneity compromised the power of their findings, and the controversy surrounding active duration of analgesia provided by IA bupivacaine was ignored because of the lack of distinction among VAS scores at different follow-up time points. Finally, these researchers did not explore the effect of concentration, dose, and epinephrine use on the effectiveness of IA bupivacaine and were thus unable to determine whether the analgesic effect of IA bupivacaine was dose-dependent.

Therefore, considering the methodological deficiencies hindering exact interpretation in many studies, we conducted a meta-analysis with the following goals: 1) to assess the effectiveness and active duration of analgesia provided by single-administration IA bupivacaine for pain relief following arthroscopic knee surgery; 2) to evaluate the effects of factors such as bupivacaine concentration and supplemental epinephrine on the analgesic effect of IA bupivacaine; 3) to determine whether there is a dose–response relationship of IA bupivacaine and analgesic effect; and 4) to assess the safety of single-administration IA bupivacaine.

## Methods

### Search strategy

This study was conducted in accordance with the Preferred Reporting Items for Systematic Reviews and Meta-Analyses (PRISMA) statement [12], an additional file shows this in more detail [see Additional file 1]. PubMed, the Cochrane Library, EMBASE, BIOSIS and Ovid databases through April 10, 2014, were searched to identify RCTs investigating a single administration of bupivacaine and using a control group.

### Study selection

Trials were included if they met the following criteria for participants, interventions, comparisons, outcomes, and study design (PICOS) criteria: 1) participants: demographically similar patients undergoing arthroscopic knee surgery; 2) intervention: single administration of IA bupivacaine after arthroscopic knee surgery; 3) comparison: placebo *vs.* no intervention; 4) outcomes: one or more of the following outcomes reported: postoperative VAS score, number of patients requiring supplementary analgesia, time to first analgesic request, and side effects; and 5) study design: RCT.

### Data extraction

Articles were reviewed and cross-checked independently by two authors (attending physicians in sports medicine). The following data available for meta-analysis were extracted: first author; country and year of publication; number and characteristics of patients; doses, concentration, and administration method of bupivacaine; epinephrine use and tourniquet use; type of anesthesia and surgery; study design; and outcomes. These data were entered into a standardized data-collection form that had been designed to receive the extracted RCT data that would be presented in Table 1. If there were more than two arms in a study, only data from the bupivacaine and placebo (or no-intervention) groups were extracted. A study was treated as two trials if it contained two independent strata. Data were extracted from histograms using Engauge Digitizer 4.0 (Free Software Foundation, Boston, MA, USA) if not provided by an article [13]. Data reported as median and range, size of a trial, mean deviation, and standard deviation were calculated using the method of Hozo [14].

**Table 1:** **Main characteristics of RCTs included in the meta-analysis**

Study (Year)	Country	Sample Size	No. of Pts (BG/PG)	Gender (M/F)	Age (yr)	Doses (mg)	Concentration (%)	Type of anaesthesia	Epinephrine	Surgery time (min)		Injection time	Tourniquet	Study design	OCEBM (R/C/B/F)
										BG	PG				
Aasbo (1996)	Norway	54	27/27	34/20	39	50	0.25	GA	No	32 ± 20	28 ± 12	8 min before RT	Yes	Double-blind	5 (1/1/2/1)
														RCT	
Alagol (2005)	Turkey	50	25/25	33/17	51.7	100	0.5	GA	No	83.8 ± 13.9	79.9 ± 10.6	15 min before RT	Yes	Double-blind	5(1/1/2/1)
														RCT	
Ates (1994)	Turkey	20	10/10	17/3	24.8	50	0.5	SA	No	NA		5 min before RT	Yes	Double-blind	5(1/1/2/1)
														RCT	
Bjornsson (1994)	Sweden	38	19/19	27/11	32	50	0.25	GA	No	27.3 ± 12	24.7 ± 10	5-10 min before RT	Yes	Double-blind	5(1/1/2/1)
														RCT	
Calmet (2004)	Spain	40	20/20	NA	NA	25	0.25	GA	No	NA		Before RT	Yes	Double-blind	5(1/1/2/1)
														RCT	
Campo (2012)	Netherlands	190	94/96	85/105	50.1	50	0.5	GA	No	17 (8–40)	16 (4–36)	Before RT	Yes	Double-blind	7(2/2/2/1)
														RCT	
Cepeda (1997)	U.K.	56	27/29	37/19	37.6	100	0.5	GA	Yes	51.5 ± 19.4	53.0 ± 19.8	5 min before RT	Yes	Double-blind	5(1/1/2/1)
														RCT	
Cook (1997)	Turkey	42	21/21	36/6	34.5	100	0.25	GA	No	NA		10 min before RT	Yes	Double-blind	7(2/2/2/1)
														RCT	
Datta (2004)	India	32	16/16	27/3	30	100	0.5	SA	No	72 ± 18	66 ± 6	Before RT	Yes	Double-blind	5(1/1/2/1)
														RCT	
De Andres (1998)	Spain	50	25/25	31/19	36.9	50	0.25	GA	No	50.2 ± 8.2	48.6 ± 12.4	10 min before RT	Yes	Double-blind	5(1/1/2/1)
														RCT	
Eroglu (2010)	Turkey	39	19/20	24/15	38.0	50	0.5	SA	No	59 ± 12	52 ± 16	10 min before RT	Yes	RCT	5(2/2/0/1)
Geutjens (1994)	U.K.	44	23/21	NA	NA	50	0.5	GA	No	NA		5 min before RT	Yes	Double-blind	4(1/0/2/1)
														RCT	
Haynes (1994)	U.K.	20	10/10	NA	NA	100	0.25	GA	Yes	NA		Before RT	Yes	Double-blind	5(1/1/2/1)
														RCT	
Henderson (1990)	USA	100	51/49	56/44	29.5	75	0.25	GA	No	45 ± 19	52 ± 24	End of surgery	Yes	Double-blind	5(1/1/2/1)
														RCT	
Izdes (2003)	Turkey	60	30/30	35/25	39.7	62.5	0.25	GA	No	33 ± 9	33 ± 9	10 min before RT	Yes	Double-blind	5(1/1/2/1)
										21 ± 3	21 ± 4			RCT	
Marchal (2003)	Spain	33	17/16	30/3	33.0	62.5	0.25	GA	Yes	72.2 ± 52.9	67.5 ± 53.3	10 min before RT	Yes	Double-blind	5(1/1/2/1)
										59.6 ± 26.8	61 ± 28.1			RCT	
Marret (2005)	France	30	15/15	17/13	48.5	150	0.5	GA	No	32 ± 6	34 ± 6	5 min before RT	Yes	Double-blind	7(2/2/2/1)
														RCT	
Milligan (1988)	U.K.	39	24/15	NA	NA	50	0.25	GA	No	NA		End of surgery	NA	Double-blind	5(1/1/2/1)
						100	0.5							RCT	
Osborne (1993)	Australia	78	38/40	55/23	33.4	100	0.5	GA	No	39.7	39.2	End of surgery	NA	Double-blind	6(1/2/2/1)
		80	40/40	60/20	34.3	100	0.5	GA	Yes	42.3	39.2			RCT	
Raja (1992)	USA	31	15/16	NA	45	50	0.25	SA	Yes	90 ± 6	90 ± 6	Before RT	Yes	Double-blind	6(1/2/2/1)
														RCT	
Smith (1991)	USA	97	49/48	65/32	35.5	150	0.5	GA	No	53 ± 19	56 ± 16	End of surgery	Yes	Double-blind	7(2/2/2/1)
														RCT	
Toivonen (2002)	Finland	120	60/60	69/51	43.1	100	0.5	GA	Yes	26 ± 14	28 ± 10	End of surgery	NA	Double-blind	7(2/2/2/1)
														RCT	
Varrassi (1999)	Italy	24	12/12	15/9	39	50	0.25	GA	No	29 ± 8	34 ± 20	10 min before RT	Yes	Double-blind	6(1/2/2/1)
														RCT	
Elsharnouby (2008)	Egypt	54	27/27	48/6	NA	50	0.25	GA	No	40 ± 5	38 ± 5	10 min before RT	Yes	Double-blind	5(1/1/2/1)
														RCT	
Karamanlioglu (2005)	Turkey	41	21/20	20/21	40.0	100	0.5	GA	No	85.2 ± 27.3	88.5 ± 26.6	End of surgery	NA	Double-blind	5(1/1/2/1)
														RCT	
Tetzlaff (1999)	USA	20	10/10	NA	NA	150	0.25	GA	Yes	NA		Before RT	NA	RCT	4(1/0/2/1)
Shaw (1997)	U.K.	58	31/27	51/7	36.3	100	0.5	GA	No	40.0	33.0	5 min before RT	Yes	Double-blind	6(1/2/2/1)
														RCT	
Chan (1995)	Singapore	20	10/10	20/0	NA	50	0.25	GA	No	NA		5 min before RT	Yes	Double-blind	4(1/0/2/1)
														RCT	

BG: Bupivacaine group; F: Female; GA: General anesthesia; M: male; mOCEBM: Modified presentation of the Oxford Centre for Evidence-Based Medicine levels of evidence; NA: Not available; PG: Placebo group; Pts: Patients; R/C/B/F: randomization/concealment of allocation/double-blinding/follow-up of patients; RCT: Randomized controlled trial; RT: Release of the tourniquet; SA: Spinal anesthesia.

### Quality assessment

The quality of each trial was evaluated by assigning a modified Oxford Center for Evidence-Based Medicine (OCEBM) grading-system score [15]. Each RCT was independently assessed by two reviewers, who were blinded to the basic information of each article, including the names of the journal and the authors, to prevent unnecessary bias. Disagreements were resolved by discussion, and a third reviewer’s opinion was asked for when necessary.

### Statistical analysis

The outcome measures were pooled using a random-effects model. For studies reporting multiple treatment groups (such as bupivacaine at different doses), each group was regarded as a single study in the meta-analysis. Stratified analysis was conducted using VAS scores at different postoperative time points (2, 4, 6, 12, 24, 48, and 72 h). Heterogeneity was assessed with Cochran’s Q statistic and quantified using the I^2^ statistic, which indicated the proportion of variability across studies. Studies with an I^2^ statistic of 25–50% are considered to have low heterogeneity, those with an I^2^ statistic of 50–75% to have moderate heterogeneity, and those with an I^2^ statistic of >75% to have a high degree of heterogeneity [16]. The effect of individual studies on the pooled effect size was assessed with a sensitivity analysis, in which the analysis was repeated omitting one study at a time, to determine the contribution of each study to the effect size.

Meta-regression models (*P ≤* 0.1 was considered statistically significant) and subgroup analyses were carried out to assess the effect of various pre-specified treatment factors (e.g., type of anesthesia, concentration of bupivacaine, epinephrine use, and tourniquet use) on treatment efficacy [17]. In the dose–response analysis, we conducted a meta-regression analysis of study-specific risk ratios (RRs) by means of weighted linear regression [18] to determine whether higher doses are associated with increased treatment effect (i.e., reduction in number of patients requiring supplementary analgesia).

*Publication bias* occurs when there is systematic underrepresentation of a given population in the published literature. Potential publication bias was assessed by visually inspecting a Begg funnel plot in which the risk ratio reported for a study was plotted against the standard error. The presence of publication bias was also evaluated by using Begg and Egger tests [19,20].

A *P* value less than 0.05 was considered statistically significant except where otherwise specified. All statistical analyses were performed using Stata Statistical Software: Release 11 (StataCorp LP, College Station, TX, USA).

## Results

### Study selection

**Figure 1:**
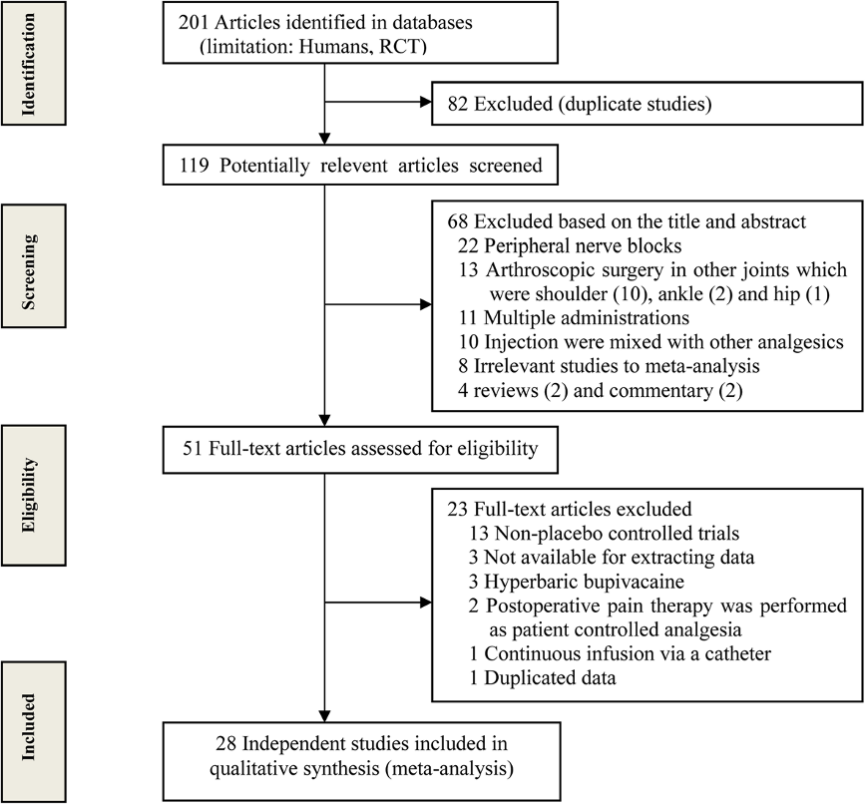
**Flowchart showing the selection of studies for meta-analysis.**

An initial database search using our search strategy identified 201 RCTs, of which 150 were excluded for being duplicate studies or for various other reasons based on the titles and abstracts. The remaining 51 full-text articles were retrieved for more detailed evaluation, and 23 were subsequently excluded. Ultimately, 28 RCTs met our inclusion criteria and were included in the present meta-analysis (Figure 1).

### Study characteristics and quality assessment

Twenty-eight independent RCTs published between 1988 and 2012, including 1,560 individuals (786 in the bupivacaine group and 774 in the placebo group), were identified [2,3,5-7,9,21-43]. The characteristics and quality assessment of the included studies are listed in Table 1. An additional file shows study characteristics and quality assessment in more detail [see Additional file 2]*.*

### Primary outcome: VAS score for pain intensity

**Figure 2:**
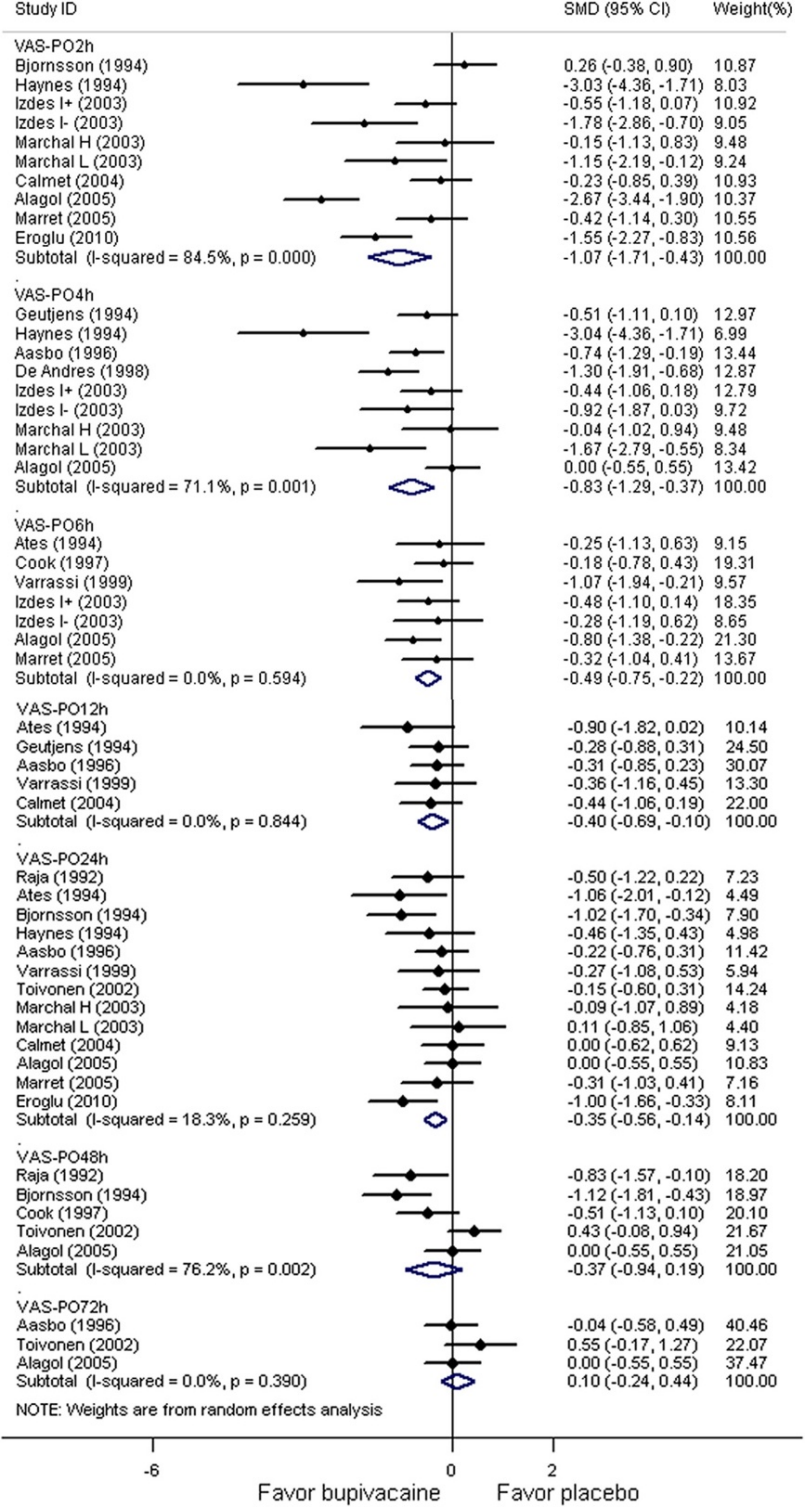
**Forest plot of meta-analysis: VAS scores (0–10 points) representing postoperative pain intensity at different time points.** VAS: visual analog scale.

Twenty-one RCTs reported postoperative VAS scores for pain intensity at different time points. Figure 2 shows the results of stratified analysis according to VAS score at different follow-up time points from the random-effects model. VAS scores were significantly lower at 2, 4, 6, 12, and 24 h in the bupivacaine group than in the placebo group. There were no significant between-group differences in postoperative VAS score at 48 h and 72 h. The association between VAS scores and follow-up time points is presented in Appendix 1 under *Association between SMD of the VAS scores and follow-up time points.*

**Figure 3:**
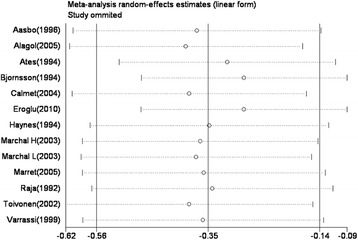
**Influence of removing studies one by one on adjusted effect estimates for 24-h-postoperative VAS scores.** Circles are effect estimates and horizontal dotted lines are 95% confidence intervals for meta-analysis of the remaining studies; the vertical center line is the pooled effect estimate for all studies.

To explore the effect of individual studies on the pooled effect size, we then conducted a sensitivity analysis for postoperative VAS score by omitting one study at a time and calculating the pooled standardized mean differences (SMDs) for the remaining studies, and found that there were no changes in the direction of effect when any one study was excluded (Figure 3).

### Secondary outcomes

Single-administration bupivacaine is associated with decreases in the number of patients requiring supplementary analgesia postoperatively and increases in the time interval before the first request for any analgesic medication. An additional file shows secondary outcomes in more detail [see Additional file 2].

### Treatment factors affecting outcomes of IA bupivacaine administration

**Figure 4:**
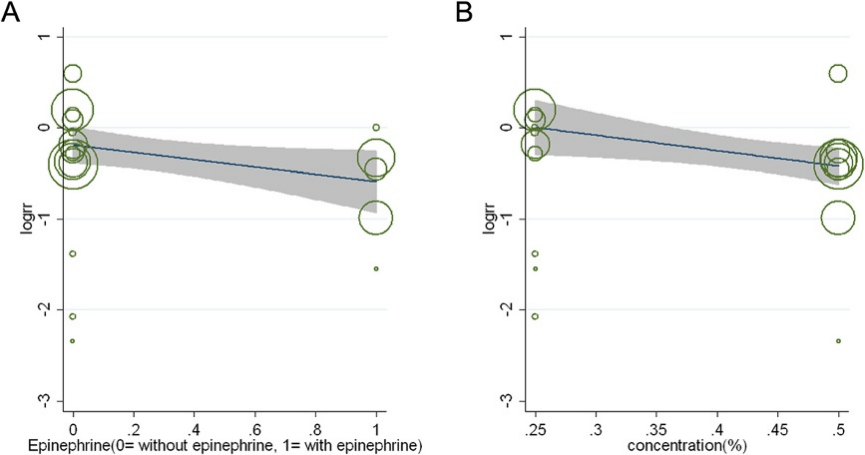
**Meta-regression analyses of (A) use of supplemental epinephrine and (B) concentration of bupivacaine.** Size of the circles corresponds to the weight of each study.

We used meta-regression analysis to evaluate the effects of various factors on outcomes and noted no association of treatment effect with type of anesthesia (coefficient, −1.69; *P* = 0.131; adjusted R^2^, 1.03%). We did, however, find associations with supplementation/no supplementation with epinephrine (coefficient, −0.412; *P* = 0.07 < 0.1; adjusted R^2^, 36.13%) and with concentration of bupivacaine (coefficient, −1.36; *P* = 0.10; adjusted R^2^, 48.43%), correlations that may have clinical implications (Figure 4). An additional file shows that effects of epinephrine use and concentration of bupivacaine on the outcomes of IA administration of bupivacaine in more detail [see Additional file 2].

### Dose–response effect of bupivacaine

**Figure 5:**
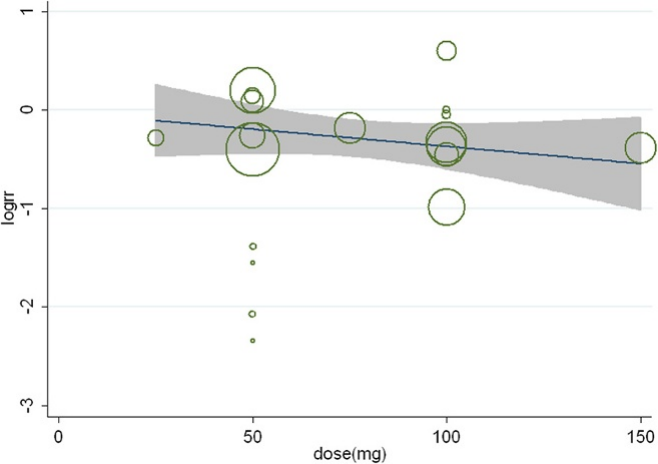
**Dose–response effect of bupivacaine.** Log of the relative risk of number of patients requiring supplementary analgesia in 19 trials of bupivacaine versus placebo by bupivacaine dose, together with a summary random-effects meta-regression. The area of each circle is inversely proportional to the variance of the log of the relative-risk estimate.

After evaluating the dose–response relationship using a single meta-regression of bupivacaine dose for treatment effect, we observed no significant relationships between reduction in the number of patients requiring supplementary analgesia and increasing bupivacaine dose (coefficient, −0.002; 95% confidence interval [CI] −0.009 to 0.005; *P* = 0.505) (Figure 5).

### Side effects

**Figure 6:**
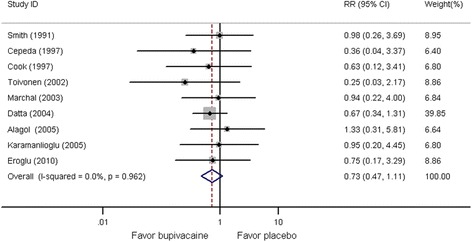
**Forest plot of meta-analysis.** Side effects.

Side effects of nausea, vomiting, urinary retention, sedation, headache, rash, and respiratory depression were evaluated in nine studies [2,23-30,34,35]. Nine studies provided data on nausea, seven provided data on vomiting, three provided data on urinary retention, two provided data on sedation, two provided data on headache, two provided data on rash, and one provided data on respiratory depression. Other complications, including postoperative bradycardia, postoperative hypotension, transient neurological symptoms, and hemarthrosis, were also reported. Figure 6 shows that there was no significant difference in person-time between participants who received IA bupivacaine and those who received placebo (RR, 0.73; 95% CI, 0.47–1.11; *P* = 0.142), and substantial heterogeneity was not seen (*P* = 0.962; I^2^ = 0%).

### Publication bias

**Figure 7:**
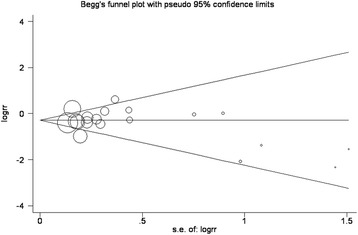
**Tests for publication bias for relative risk of the number of patients requiring supplementary analgesia.**

There was no potential publication bias among the included trials (Egger’s test, *P* = 0.548; Begg’s test, *P* = 0.529, Figure 7).

## Discussion

The present meta-analysis of 28 RCTs [2,3,5-7,9,21-43] evaluating the safety and efficacy of single-administration IA bupivacaine in the management of pain after arthroscopic knee surgery has clearly shown that a single administration of IA bupivacaine is significantly better than placebo and blank intervention at relieving pain. Moreover, it may decrease the number of patients requiring supplementary analgesia and prolong the time to first request for analgesia. The most important findings of the present study are that 1) the analgesic effect could be associated with concomitant epinephrine and with concentration of bupivacaine, 2) there was no dose–response relationship of single-administration IA bupivacaine on the analgesic effect, and 3) the use of single-administration IA may not increase the prevalence of side effects during short-term observation.

With regard to duration of analgesic effect, our meta-analysis showed that single-administration IA bupivacaine is effective for pain management for approximately 24 h following arthroscopic knee surgery, which is consistent with some previous studies [22,25,32,42]. However, most of the RCTs [3,5,7,9,21,23,24,26-28,30,31,33,36-38,40,41] included in this analysis reported either no effect or only a very short (less than 12-hour) effect duration of single-administration IA bupivacaine compared with placebo. Various factors may have been responsible for the conflicting results regarding the use of IA bupivacaine for pain control after arthroscopic knee surgery. Various acute or chronic comorbidities might have had led to different levels of pain tolerance [3], and the type of anesthetic (general and/or spinal anesthetic) patients received [29] might have affected the results because different amounts of intraoperative opioids were used. The use of pre- and/or perioperative opioids might also have influenced postoperative analgesia [21]; for example, augmentation with epinephrine might have slowed the release of analgesia into the vascular system. In addition, postoperative hemarthrosis may have increased the level of pain and decreased the concentration of anesthetic within the knee joint [43]. Concerns about the concentration and duration of action of bupivacaine have also been reported [41]. However, despite all these confounders, the overall results of our meta-analysis suggest that the patients receiving IA bupivacaine had an analgesic effect for 24 h postoperatively, and analysis of postoperative time points revealed a positive correlation. (An additional file shows that in more detail [see Additional file 3: Figure S1]). In other words, the absolute difference of VAS pain score (SMD) between the bupivacaine group and the placebo group decreased as time progressed postoperatively. Decreasing pain intensity over the follow-up period could also explain this result.

In the present meta-analysis, we assessed the effects of various pre-specified treatment factors on the treatment efficacy of IA bupivacaine. According to our results, the analgesic effect of IA bupivacaine appears to be associated with epinephrine use and concentration of bupivacaine, findings that may be applicable to clinical practice. These findings are supported by some early research [5,9,29]. As a concomitant treatment, epinephrine is considered to improve the efficacy of a local anesthetic by slowing its release into the vascular system, and local presence of epinephrine may alter the inflammatory process, thereby interfering with the activation of the opiate receptors [5]. Epinephrine may also protect from systemic toxicity [44]. The concentration of bupivacaine also affected the results. A higher concentration of bupivacaine traverses the synovium more rapidly to reach the joint capsule, which is perforated by articular vessels and nerve endings [3]; indeed, in the present analysis a 0.5% concentration of bupivacaine was associated with a better analgesic effect than 0.25%, which supports the use of a higher concentration of bupivacaine. In general, the administration of IA 0.5% bupivacaine augmented by epinephrine in clinical practice may be advisable.

The present meta-analysis investigated whether the effectiveness of IA bupivacaine after arthroscopic knee surgery was dose-dependent. Our results suggest that there was no association between the reduction in number of patients requiring supplementary analgesia and bupivacaine dose, a potentially valuable finding given that high doses of IA bupivacaine may be associated with adverse side effects. Recent studies have demonstrated dose dependent chondrotoxic effects of bupivacaine *in vitro* as well as *in vivo* [45,46], suggesting that low-dose IA bupivacaine is potentially the least harmful strategy [47]. Despite quite strong evidence for chondrotoxicity, the incidence of chondrolysis following IA administration of bupivacaine in clinical practice seems to be low or possibly underreported [48]. Given equal efficacy for pain control after arthroscopic knee surgery across doses and a dose–response relationship for chondrotoxic effects, a clinical decision leaning toward low-dose (50-mg) bupivacaine appears to be supported, although the lowest effective bupivacaine dose has not yet been identified.

Our meta-analysis revealed no significant difference in the rate of side effects between the IA-bupivacaine group and the placebo group. Consistent with a previous review [1], this important finding establishes the safety of IA bupivacaine during very short term observation. Moreover, compared with continuous IA infusion of analgesics, which is associated with large effusion of the surgical wound and direct access for infectious agents with catheter placement, single-administration IA bupivacaine maximizes the safety of postoperative pain relief in the early postoperative period. Nevertheless, it should be recognized that the follow-up period in the majority of studies was not long enough to detect signs and symptoms of infection. Although plasma levels were all below reported toxic plasma bupivacaine concentrations [49], data on cardiac and central nervous system toxicity of megadose bupivacaine for assessment of long-term safety are lacking. Further investigation of the long-term safety of IA bupivacaine is therefore required.

The present study has some limitations that should be taken into account. First, we acknowledge that the individual studies included relatively small numbers of patients (n < 30 in three studies [30,37,40]) and that overestimation of the treatment effect is more likely in smaller trials. Second, there was considerable heterogeneity among the included trials. Lack of standardization of IA-bupivacaine administration (e.g., with respect to injection time) and differing study designs may have led to heterogeneity and potentially affected our results. Furthermore, side effects require some time to become apparent, but none of our included studies had long enough observation periods to accomplish this.

## Conclusions

In conclusion, current evidence suggests that single-administration IA bupivacaine is effective for pain relief after arthroscopic knee surgery. Low-dose administration of IA bupivacaine 0.5% combined with epinephrine may be advisable in clinical practice. However, because the follow-up period in majority studies may not have been of sufficient duration, more safety data during long-term follow-up are required.

## Additional files

Additional file 1:
**PRISMA 2009 Checklist.**
Additional file 2: **1) Study characteristics and quality assessment.** 2) Association between SMD of the VAS scores and follow-up time points. 3) Secondary outcomes. 4) Effects of epinephrine use and concentration of bupivacaine on the outcomes of IA administration of bupivacaine. Additional file 3: Figure S1. Association between difference in VAS scores (SMD) for pain intensity and follow-up time points. **Figure S2.** Forest plot of meta-analysis: number of patients requiring supplementary analgesia. **Figure S3.** Influence of removing studies one by one on adjusted effect estimates of patients requiring supplementary analgesia. Circles are effect estimates and horizontal dotted lines are 95% confidence intervals for meta-analysis of the remaining studies; the center vertical line is the pooled effect estimate for all studies. **Figure S4.** Forest plot of meta-analysis: time to first request for analgesia. **Figure S5.** Forest plot of subgroup meta-analysis: treatment effects with/without epinephrine. **Figure S6.** Forest plot of subgroup meta-analysis: treatment effects with two concentrations (0.25% and 0.5%) of bupivacaine.
