# Supplementary material for: Single administration of intra-articular bupivacaine in arthroscopic knee surgery: a systematic review and meta-analysis
Source: BMC Musculoskelet Disord. 2015 Feb 10;16:21. doi: 10.1186/s12891-015-0477-6 (PMC4328055; doi:10.1186/s12891-015-0477-6)
Supplement: Additional file 2: — 1) Study characteristics and quality assessment. 2) Association between SMD of the VAS scores and follow-up time points. 3) Secondary outcomes. 4) Effects of epinephrine use and concentration of bupivacaine on the outcomes of IA administration of bupivacaine. [file 12891_2015_477_MOESM2_ESM.doc]

**Additional file 2**

**Study characteristics and quality assessment**

Twenty studies were based in Europe, four in North America, two in Asia, one in Oceania, and one in Africa. No studies were based in South America. Sample sizes ranged from 20 to 190. Consistent baselines were observed for all patients. Patients in all studies received single-administration IA bupivacaine after arthroscopic knee surgery, and all studies had a control group. One study [27], in which there were two independent strata (high-inflammatory and low-inflammatory groups), was treated as two trials. One study [26] had inflammation-positive and inflammation-negative subgroups in the experimental and placebo groups; its subgroups were combined and regarded as one group. Two studies [6, 7] had three-group trials: one with a placebo and two experimental groups (high and low doses of bupivacaine and one with and without epinephrine) that were analyzed separately and therefore treated as two studies. As a result, the placebo group in each of these studies was counted twice.

Of the 28 eligible papers, 25 were high-quality RCTs. The quality score of each individual study was calculated as the average value of the MOS scores given by the two assessors. MOS scores ranged from 4 to 7 points (mean, 5.0). Percent agreement between the two assessors’ quality reviews ranged from 88% to 100%, indicating good agreement between reviewers in quality assessment.

**Association between SMD of the VAS scores and follow-up time points**

A positive correlation was found between SMDs of the VAS scores and follow-up (r = 0.69, *P*= 0.02) and is depicted in Figure S1.

**Secondary outcomes**

Single-administration bupivacaine is associated with decreases in number of patients requiring supplementary analgesia postoperatively. Figure S2 shows that the number in the bupivacaine group was 0.82 times that of the placebo group (17 RCTs; RR, 0.75; 95% CI 0.61–0.93; *P*= 0.007). There was moderate heterogeneity (*P*= 0.001, I^2^ = 56.5%). We also conducted a sensitivity analysis by omitting one study at a time. Figure S3 shows that there were no changes in the direction of effect when any one study was excluded.

Twelve RCTs reported data on the time interval before the first request for any analgesic medication. Figure S4 shows a significant difference in time (SMD, 2.95; 95% CI, 1.82–4.08; *P*= 0.000). Time to first request for analgesia had a high degree of heterogeneity (*P*= 0.000; I^2^ = 96.5%), but upon sensitivity analyses, the results did not change with the exclusion of any single study, and the overall SMD ranged from 2.08 (95% CI, 1.07–3.09; *P*= 0.000) to 3.36 (95% CI, 2.09–4.62; *P*= 0.000).

**Effects of epinephrine use and concentration of bupivacaine on the outcomes of IA administration of bupivacaine**

Subgroup analyses were conducted for use of epinephrine and concentration of bupivacaine. Figure S5 shows that single-administration bupivacaine in combination with epinephrine was associated with a 25% improvement in treatment effect (reduction in number of patients requiring supplementary analgesia). Figure S6 shows that a 0.5% concentration of bupivacaine was associated with a 21% improvement in treatment effect compared with bupivacaine 0.25% (Figure S6).
